# Association Factor for Identifying Linear and Nonlinear Correlations in Noisy Conditions

**DOI:** 10.3390/e22040440

**Published:** 2020-04-13

**Authors:** Nezamoddin N. Kachouie, Wejdan Deebani

**Affiliations:** 1 Department of Mathematical Sciences, Florida Institute of Technology, Melbourne, FL 32901, USA; 2 Deparments of Mathematics, College of Science and Arts, King Abdulaziz University, P.O. Box 344, Rabigh 21911, Saudi Arabia; wdeebani@kau.edu.sa

**Keywords:** association factor, Pearson’s correlation, distance correlation, maximal information coefficient (MIC), detrended fluctuation analysis (DFA), nonlinear relation, noisy relationship

## Abstract

Background: In data analysis and machine learning, we often need to identify and quantify the correlation between variables. Although Pearson’s correlation coefficient has been widely used, its value is reliable only for linear relationships and Distance correlation was introduced to address this shortcoming. Methods: Distance correlation can identify linear and nonlinear correlations. However, its performance drops in noisy conditions. In this paper, we introduce the Association Factor (AF) as a robust method for identification and quantification of linear and nonlinear associations in noisy conditions. Results: To test the performance of the proposed Association Factor, we modeled several simulations of linear and nonlinear relationships in different noise conditions and computed Pearson’s correlation, Distance correlation, and the proposed Association Factor. Conclusion: Our results show that the proposed method is robust in two ways. First, it can identify both linear and nonlinear associations. Second, the proposed Association Factor is reliable in both noiseless and noisy conditions.

## 1. Introduction

Analyzing large datasets is becoming central in science, engineering, and technology. In data mining and statistical analysis, it is essential to detect relationships between different variables [1]. Different correlation factors have been introduced to identify and quantify the relationship between variables. Pearson’s correlation coefficient has been broadly used to identify and measure the strength and direction of a linear relationship between two variables.

Pearson’s correlation can effectively detect linear relationships; however it is not reliable to identify nonlinear relationships between two variables. To address this shortcoming of Pearson’s correlation, Distance correlation was introduced by Gábor J. Székely [2,3] to find both linear and nonlinear relationships between two variables. Regardless of the relationship type, Distance correlation quantifies the degree of correlation by a value between zero and one. Values close to one represent strong correlation, while values close to zero suggest weak correlation between two variables. It has been demonstrated that Distance correlation is superior to Pearson’s correlation for identifying nonlinear relationships.

Several extensions of Distance correlation have been introduced such as Invariant Distance correlation [4], Conditional Distance correlation [5], Distance Correlation of Lancaster distributions [6], Distance Standard Deviation [7], Distance Correlation for locally stationary processes [8], Distance correlation coefficient for multivariate functional data [9], Partial Distance correlation [10], and Distance correlation *t*-test [11]. Distance correlation has been broadly used for different applications such as time series [12,13], clinical data analysis [14], genomics [15], and biomedical data analysis [16].

Although Distance correlation can identify and quantify nonlinear correlations, it does not necessarily obtain the same or comparable values for different nonlinear relationships. For example, the Distance correlation of an exponential relationship could be higher than a quadratic relationship. Moreover, Distance correlation values drop in noisy conditions and may not robustly demonstrate the strength of the correlation, where low correlation values may contribute to the wrong conclusion about the strength of relationship between two variables.

To address these shortcomings and improve the performance of Distance correlation, in this paper we propose the Association Factor (AF). The proposed AF performs robustly with regard to identifying both linear and nonlinear relationships. Moreover, we show that AF performs robustly in noisy conditions and outperforms Distance correlation in identifying noisy linear and nonlinear relationships. An overview of Pearson’s correlation and Distance correlation is provided in the next section. The proposed Association Factor is presented in Section 2. Simulation models, Results, and Conclusions are presented in Section 3, Section 4 and Section 5 respectively.

## 2. Methods

### 2.1. Quick Review

#### 2.1.1. Pearson’s Correlation

Pearson’s correlation is a measure of the strength and direction of the linear relationship between two variables. Its score ranges between −1 and one, and it describes the degree to which one variable is linearly related to another. Pearson’s correlation between two variables X and Y is defined by:

(1) $$\rho =\frac{cov(X,Y)}{\sigma_{X}\sigma_{Y}}$$

where cov(X,Y) is the covariance between X and Y, $\sigma_{X}$ is the standard deviation of X, and $\sigma_{Y}$ is the standard deviation of Y. Pearson’s correlation is essentially the covariance of X and Y normalized by the product of the standard deviations of X and Y [17].

#### 2.1.2. Distance Correlation

Distance correlation is a measure of the correlation between two random vectors *X* and *Y*, and its value ranges from zero to one. Analogous to product-moment correlation (Pearson’s correlation), Distance correlation can identify linear and nonlinear correlations using Euclidean distance. The empirical Distance correlation [2] is computed by:

(2) $$R^{2}\left(X,Y\right)=\left\{\begin{array}{cc} \frac{\nu_{n}^{2}\left(X,Y\right)}{\sqrt{\nu_{n}^{2}\left(X\right)\nu_{n}^{2}\left(Y\right)}}, & \nu_{n}^{2}\left(X\right)\nu_{n}^{2}\left(Y\right)>0,\\ 0, & \nu_{n}^{2}\left(X\right)\nu_{n}^{2}\left(Y\right)=0 \end{array}\right.$$

where R(X,Y) is empirical Distance correlation, $\nu_{n}\left(X,Y\right)$ is empirical Distance covariance of *X* and *Y*, $\nu_{n}\left(X\right)$ and $\nu_{n}\left(Y\right)$ are empirical Distance variances of *X* and *Y* respectively, *n* is sample size, and $\nu_{n}\left(.,.\right)$ is a scalar. R(X,Y) is zero if and only if *X* and *Y* are independent.

### 2.2. Proposed Association Factor

In this paper, we introduce the Association Factor (AF), the Distance correlation of Optimal Transformations of variables X and Y:

(3) $$R_{AF}\left(X,Y\right)=R\left(h_{1}\left(X\right),h_{2}\left(Y\right)\right)$$

where $R_{AF}\left(X,Y\right)$ is the proposed AF, and $h_{1}:dom\left(X\right)\to B$ and $h_{2}:dom\left(Y\right)\to C$ are measurable mean zero transformations where B,C⊆R, and for $\nu_{n}^{2}\left(h_{1}\left(X\right)\right)\nu_{n}^{2}\left(h_{2}\left(Y\right)\right)>0$, we have:

(4) $$R_{AF}^{2}\left(X,Y\right)=\frac{\nu_{n}^{2}\left(h_{1}\left(X\right),h_{2}\left(Y\right)\right)}{\sqrt{\nu_{n}^{2}\left(h_{1}\left(X\right)\right)\nu_{n}^{2}\left(h_{2}\left(Y\right)\right)}}$$

where $\nu_{n}\left(h_{1}\left(X\right),h_{2}\left(Y\right)\right)$ is empirical Distance covariance of $h_{1}\left(X\right)$ and $h_{2}\left(Y\right)$, $\nu_{n}\left(h_{1}\left(X\right)\right)$ and $\nu_{n}\left(h_{2}\left(Y\right)\right)$ are empirical Distance variances of $h_{1}\left(X\right)$ and $h_{2}\left(Y\right)$ respectively, *n* is sample size, and $\nu_{n}\left(.,.\right)$ is a scalar:

(5) $$\nu_{n}^{2}\left(h_{1}\left(X\right),h_{2}\left(Y\right)\right)=\frac{1}{n^{2}}\sum_{k,l=1}^{n}A_{kl,h_{1}}B_{kl,h_{2}}$$

(6) $$\nu_{n}^{2}\left(h_{1}\left(X\right)\right)=\nu_{n}^{2}\left(h_{1}\left(X\right),h_{1}\left(X\right)\right)=\frac{1}{n^{2}}\sum_{k,l=1}^{n}A_{kl,h_{1}}^{2}$$

(7) $$\nu_{n}^{2}\left(h_{2}\left(Y\right)\right)=\nu_{n}^{2}\left(h_{2}\left(Y\right),h_{2}\left(Y\right)\right)=\frac{1}{n^{2}}\sum_{k,l=1}^{n}B_{kl,h_{2}}^{2}$$

where:

$$A_{kl,h_{1}}=a_{kl,h_{1}}-{\bar{a}}_{k.,h_{1}}-{\bar{a}}_{.l,h_{1}}+{\bar{a}}_{..,h_{1}},$$

$$a_{kl,h_{1}}={\left|h_{1}\left(X_{k}\right)-h_{1}\left(X_{l}\right)\right|}_{p},\;{\bar{a}}_{k.,h_{1}}=\frac{1}{n}\sum_{l=1}^{n}a_{kl,h_{1}},$$

$${\bar{a}}_{.l,h_{1}}=\frac{1}{n}\sum_{k=1}^{n}a_{kl,h_{1}},\;{\bar{a}}_{..,h_{1}}=\frac{1}{n^{2}}\sum_{k,l=1}^{n}a_{kl,h_{1}},$$

k,l=1,...,n

Similarly,

$$B_{kl,h_{2}}=b_{kl,h_{2}}-{\bar{b}}_{k.,h_{2}}-{\bar{b}}_{.l,h_{2}}+{\bar{b}}_{..,h_{2}},$$

$$b_{kl,h_{2}}={\left|h_{2}\left(Y_{k}\right)-h_{2}\left(Y_{l}\right)\right|}_{q},\;{\bar{b}}_{k.,h_{2}}=\frac{1}{n}\sum_{l=1}^{n}b_{kl,h_{2}},$$

$${\bar{b}}_{.l,h_{2}}=\frac{1}{n}\sum_{k=1}^{n}b_{kl,h_{2}},\;{\bar{b}}_{..,h_{2}}=\frac{1}{n^{2}}\sum_{k,l=1}^{n}b_{kl,h_{2}},$$

k,l=1,...,n

To quantify the degree of association between *X* and *Y*, we discuss a bivariate case of a response variable *Y* and a predictor *X*. Regardless of the relationship type between *X* and *Y*, we assume there are transforming functions $h_{1}\left(X\right)$ and $h_{2}\left(Y\right)$ that can transform the relationship between *X* and *Y* to a linear relation between $h_{1}\left(X\right)$ and $h_{2}\left(Y\right)$:

(8) $$h_{2}\left(Y\right)=\beta_{0}+h_{1}\left(X\right)+\epsilon$$

where ϵ has a Gaussian distribution with zero mean and standard deviation σ. We can find $h_{1}\left(X\right)$ and $h_{2}\left(Y\right)$ by minimizing the Sum of Squared Errors (SSE):

(9) $$\sum_{i=1}^{n}{\hat{\epsilon }}_{i}^{2}=\sum_{i=1}^{n}{\left(\hat{h_{2}}\left(Y_{i}\right)-\hat{h_{1}}\left(X_{i}\right)\right)}^{2}$$

To minimize $\sum_{i=1}^{n}{\hat{\epsilon }}_{i}^{2}$ with regard to $h_{1}\left(X\right)$ and $h_{2}\left(Y\right)$, we use a simplified optimal transformation [18] by an iterative estimation. Let $E[h_{2}\left(Y\right)]=0$, $Var\left[h_{2}\left(Y\right)\right]=E\left[{\left(h_{2}\left(Y\right)\right)}^{2}\right]-E{\left[h_{2}\left(Y\right)\right]}^{2}=1$, and as a result, $E[{\left(h_{2}\left(Y\right)\right)}^{2}]=1$. We start with $h_{2}\left(Y\right)=\frac{Y}{\left||Y|\right|}$. For a given $h_{2}\left(Y\right)$, to minimize $\sum_{i=1}^{n}{\hat{\epsilon }}_{i}^{2}$, we have:

(10) $$h_{1}\left(X\right)=E\left[h_{2}\left(Y\right)|X\right]$$

and for a given $h_{1}\left(X\right)$, in a similar way, we have:

(11) $$h_{2}\left(Y\right)=\frac{E[h_{1}\left(X\right)|Y]}{\left||E[h_{1}\left(X\right)|Y]|\right|}$$

In each iteration, $h_{1}\left(X\right)$ and $h_{2}\left(Y\right)$ will be estimated, and an iterative optimization continues until the estimate of error $\sum_{i=1}^{n}\epsilon_{i}^{2}\left(T\right)=\sum_{i=1}^{n}{\left(h_{2}\left(Y_{i}\right)\left(T\right)-h_{1}\left(X_{i}\right)\left(T\right)\right)}^{2}$ does not decrease in iteration *T*, where $h_{2}\left(Y\right)\left(T\right)$ and $h_{1}\left(X\right)\left(T\right)$ are optimal estimates with regard to unexplained variance.

Estimated transforming functions $h_{1}\left(X\right)$ and $h_{2}\left(Y\right)$ are optimal and linear for a joint normal distribution [19], where marginal distributions of *X* and *Y* are normal. If joint distribution of *X* and *Y* is not normal, estimated transforming functions $h_{1}\left(X\right)$ and $h_{2}\left(Y\right)$ are not optimal, but they are close to optimal linear transformations [18]. AF has the following properties:

- Non-negativity, $R_{AF}\left(X,Y\right)\geq 0$.
- Disappears if and only if the two vectors are not associated, $R_{AF}\left(X,Y\right)=0$ for unassociated *X* and *Y*.
- Symmetry, $R_{AF}\left(X,Y\right)=R_{AF}\left(Y,X\right)$ for noiseless vectors *X* and *Y*.
- Triangular inequality, $R_{AF}\left(X,Y\right)\leq R_{AF}\left(X,Z\right)+R_{AF}\left(Z,Y\right)$.

## 3. Simulation Models

To test the performance of the proposed AF, we modeled several simulations and computed Pearson’s correlation, Distance correlation, and the proposed AF. Because Distance correlation and the proposed AF take values between zero and one, we calculate the absolute value of Pearson’s correlation to provide a fair comparison between these methods. The aforementioned correlation coefficients are quantified for linear and nonlinear correlations. We have also obtained these correlation coefficients for random relation (no relationship) as follows.

### 3.1. Linear and Nonlinear Relationships in Noiseless Conditions

We simulated the following relationships:

- Linear: $Y=\beta_{0}+\beta_{1}X$, where $\beta_{0}$ is intercept and $\beta_{1}$ is slope.
- Fourth order polynomial: $Y=\beta_{0}+\beta_{1}X^{1}+\beta_{2}X^{2}+\beta_{3}X^{3}+\beta_{4}X^{4}$ where β’s are coefficients.
- Exponential: Y=exp(λx), where λ is rate.
- Parabolic: $Y=\beta_{0}{\left(X-\beta_{1}\right)}^{2}$, where $\beta_{0}$ and $\beta_{1}$ are coefficients.

The simulation steps are summarized below.

1. Let $\Omega_{D}$ be a set of *D* relationship types $l_{1}$ to $l_{D}$. Generate pairwise variables using relationships in the relationship set $\Omega_{D}$ so that *D* different datasets $\Gamma^{1},\Gamma^{2},...,\Gamma^{D}$ representing the relationship types $l_{1}$ to $l_{D}$ are obtained.
2. For each generated dataset $\Gamma^{d}$, compute Pearson’s correlation (absolute value) $\rho^{d}$, distance correlation $R^{d}$, and Association Factor $R_{AF}^{d}$.
  (12) d=1,2,...,D.

### 3.2. Linear and Nonlinear Relationships in Noisy Conditions

To test the performance of the proposed AF in noisy conditions, we corrupt the true relationships with low, medium, and high noise:

- Linear: $Y=\beta_{0}+\beta_{1}X+\epsilon_{\sigma }$.
- Fourth order polynomial: $Y=\beta_{0}+\beta_{1}X^{1}+\beta_{2}X^{2}+\beta_{3}X^{3}+\beta_{4}X^{4}+\epsilon_{\sigma }$.
- Exponential: $Y=exp\left(\lambda x\right)+\epsilon_{\sigma }$.
- Parabolic: $Y=\beta_{0}{\left(X-\beta_{1}\right)}^{2}+\epsilon_{\sigma }$.

where $\epsilon_{\sigma }$ is White (Gaussian) noise and noise level is specified by standard deviation of the Gaussian distribution (σ). We then quantify the linear and nonlinear correlations in noisy conditions using Pearson’s correlation, Distance correlation, and Association Factor. In the noisy conditions, we calculate the Monte Carlo average of each correlation coefficient over T=100 instances (trials) of the same noise level.

The simulation steps are summarized below.

1. Let $\Omega_{D}$ be a set of *D* relationship types $l_{1}$ to $l_{D}$. Generate pairwise variables using relationships in the relationship set $\Omega_{D}$ so that *D* different datasets $\Gamma^{1},\Gamma^{2},...,\Gamma^{D}$ representing the relationship types $l_{1}$ to $l_{D}$ are obtained.
2. Set t=1 (trial one).
3. Generate noisy relationships $\Phi_{t}^{1},\Phi_{t}^{2},...,\Phi_{t}^{D}$ by adding Gaussian noise (with noise level $\epsilon_{\sigma }$) to the datasets $\Gamma^{d}$’s generated using the true relationships ($l_{d}$’s).
4. Compute and save Pearson’s correlation (absolute value) $\rho_{t}^{d}$, distance correlation $R_{t}^{d}$, and association factor $R_{AF,t}^{d}$ for each noisy dataset $\Phi_{t}^{d}$.
5. Increase *t* by one (t=t+1).
6. Repeat Steps 3 to 5 while t≤T.
7. Compute the Monte Carlo average of each correlation measure as follows: $\rho^{d}=\sum_{t=1}^{T}\frac{\rho_{t}^{d}}{T},R^{d}=\sum_{t=1}^{T}\frac{R_{t}^{d}}{T},R_{AF}^{d}=\sum_{t=1}^{T}\frac{R_{AF,t}^{d}}{T}.$

### 3.3. No Relationship

We also investigated whether functions $h_{1}$ and $h_{2}$ may introduce a spurious relationship into the relationship between *X* and *Y*. To address this, we obtained Pearson’s correlation, Distance correlation, and AF for no relationship (random noise).

### 3.4. Symmetry Regarding Sample Size, Missing Data, and Noise Level

Next, we study the symmetry of AF regarding the response and factor. The goal here is to investigate whether the Association Factor quantifies the relationship between *X* and *Y* regardless of their order. This means whether $R_{AF}^{2}\left(X,Y\right)$ is equal to $R_{AF}^{2}\left(Y,X\right)$. For the true relationship without noise, we calculate $R_{AF}^{2}\left(X,Y\right)$ assuming:

(13) $$h_{2}\left(Y\right)=\beta_{0,X}+h_{1}\left(X\right)$$

and to compute $R_{AF}^{2}\left(Y,X\right)$, we have:

(14) $$h_{2}\left(X\right)=\beta_{0,Y}+h_{1}\left(Y\right)$$

For the noisy relationship, to compute $R_{AF}^{2}\left(X,Y\right)$, we assume:

(15) $$h_{2}\left(Y\right)=\beta_{0,X}+h_{1}\left(X\right)+\epsilon$$

and similarly for $R_{AF}^{2}\left(Y,X\right)$, we have:

(16) $$h_{2}\left(X\right)=\beta_{0,Y}+h_{1}\left(Y\right)+\epsilon$$

We study the symmetry of AF with regard to the sample size and noise level for nonlinear relationships. We will show that with a small sample size, the underlying relationship cannot be visually identified even in the noiseless case due to the missing data.

### 3.5. Entropic Distance

We also compute Entropic Distance (ED) and compare it with AF. Entropic Distance, also called “relative entropy”, is the differences between entropies with and without a prior condition [20]. The conditional entropy of two variables *X* and *Y* taking values *x* and *y*, respectively, is defined by:

(17) $$R_{ED}\left(X|Y\right)=-\sum_{y}p\left(y\right)\sum_{x}p\left(x|y\right)log_{b}p\left(x|y\right),$$

where *b* is the logarithm base. ED has the following properties [21]:

1. ED is symmetric.
2. ED is zero for comparing a distribution with itself.
3. ED is positive for two different distributions.

AF values are bounded between zero and one, but ED does not have an upper bound and can take any positive value. Therefore, the interpretation of ED is subjective, while AF can objectively represent the strength of the underlying relationship. Therefore, rather than comparing the ED and AF values, we computed the AF ratio and the ED ratio for different noise conditions. Let $R_{AF_{L}}^{2}$ and $R_{AF_{H}}^{2}$ be the AF for a relationship corrupted with different noise levels. The AF ratio, $I_{AF}$, is computed by:

(18) $$I_{AF}=\frac{R_{AF_{H}}^{2}}{R_{AF_{L}}^{2}}*100$$

Hence, the AF ratio can be interpreted as:

- $I_{AF}<100\%$ indicates a decrease in AF.
- $I_{AF}>100\%$ indicates an increase in AF.
- $I_{AF}=100\%$ indicates no change in AF.

### 3.6. Detrended Fluctuation Analysis (DFA)

Peng et al. introduced Detrended Fluctuation Analysis (DFA), which is commonly used in time series analysis and stochastic processes [22]. It is an alternative method in comparison with the auto-correlation function and is often used for determining the statistical self-similarity of a signal. It can detect long-range correlations in a patchy signal. The computation of DFA [22,23] is summarized below.

For a time series of total length *N*:

- Integrate the time series:
  $$y\left(k\right)=\sum_{i=1}^{k}\left[B_{i}-B_{ave}\right]$$
  where $B_{i}$ is the ith interval and $B_{avg}$ is the average interval.
- Divide the integrated time series into boxes of equal length *n*.
- Fit a line to the data in each box of size *n* separately. The *y* coordinate of the straight line segment in a box is denoted by $y_{n}\left(k\right)$.
- Remove the trend (detrend) from the integrated time series y(k) by subtracting the local trend $y_{n}\left(k\right)$ in each box.
- Calculate the root-mean-squared fluctuation, F(n), of the obtained detrended time series by:
  $$F\left(n\right)=\sqrt{\frac{1}{N}\sum_{k=1}^{N}{\left[y\left(k\right)-y_{n}\left(k\right)\right]}^{2}}$$
- Repeat this computation over all time scales (box size n) to provide a relationship between F(n) and the box size (*n*).

## 4. Simulation Results and Discussion

We compared the performance of Pearson’s correlation, Distance correlation, and the Association Factor for the following relationships that were explained in detail in the previous section:

- Linear: $Y=1+X+\epsilon_{\sigma }$.
- Fourth order polynomial: $Y=X^{4}+\epsilon_{\sigma }$.
- Exponential: $Y=exp\left(0.05x\right)+\epsilon_{\sigma }$.
- Parabolic: $Y=4{\left(X-0.5\right)}^{2}+\epsilon_{\sigma }$.

**Figure 1:**
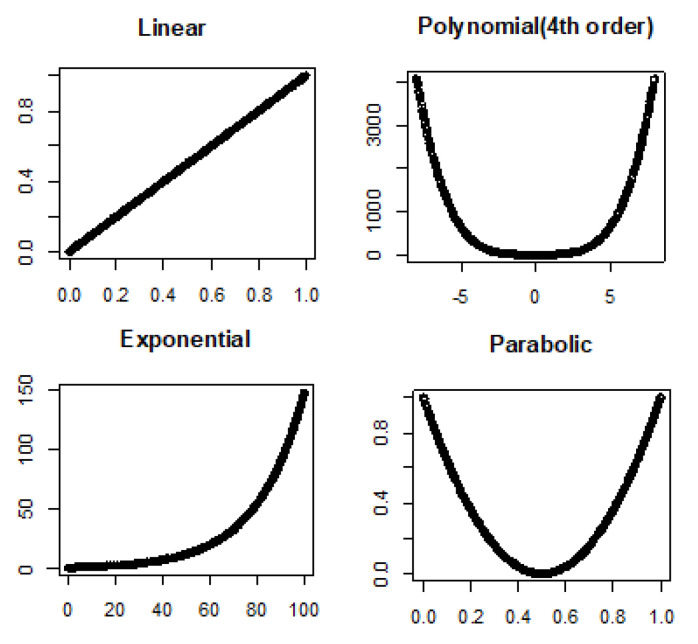
True (without noise) linear, polynomial, exponential, and parabolic relationship types.

**Figure 2:**
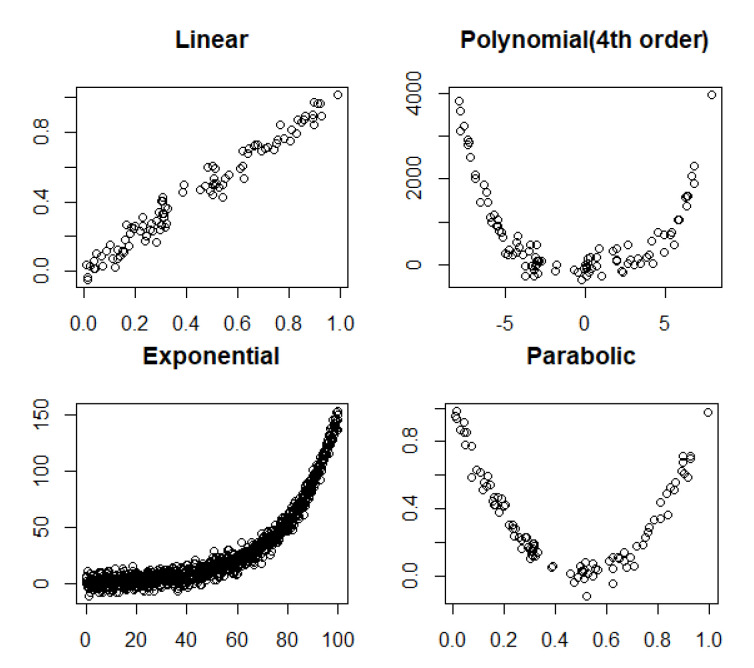
Linear, polynomial, exponential, and parabolic relationship types corrupted with low noise.

**Figure 3:**
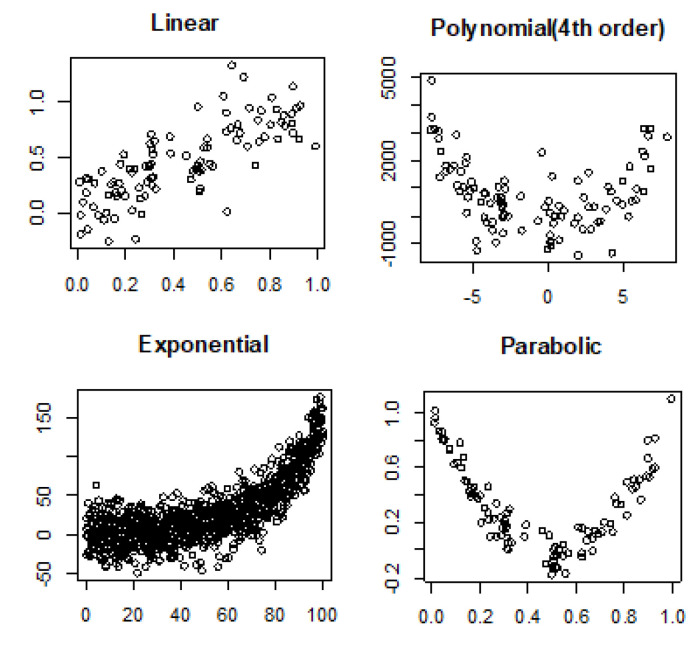
Linear, polynomial, exponential, and parabolic relationship types corrupted with medium noise.

**Figure 4:**
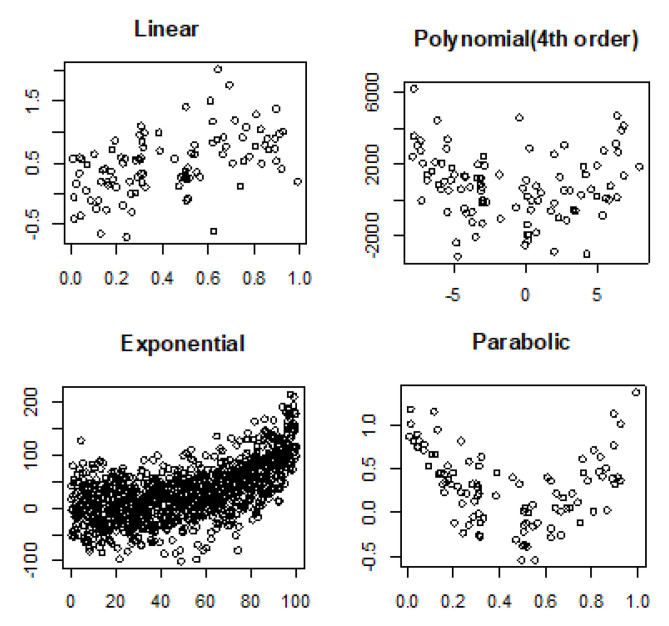
Linear, polynomial, exponential, and parabolic relationship types corrupted with high noise.

**Figure 5:**
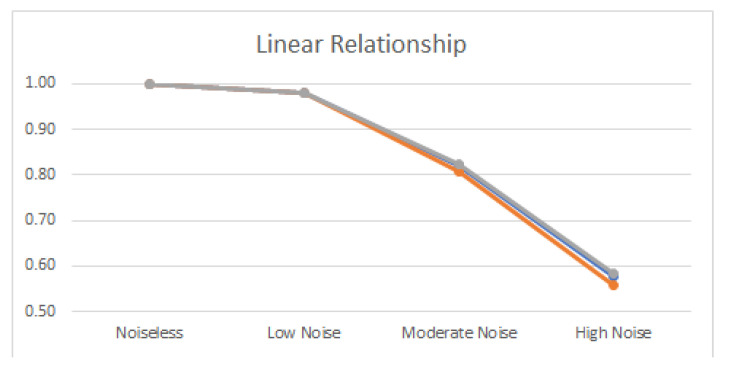
Pearson’s correlation (blue), Distance correlation (orange), and Association Factor (gray) scores for true linear relationship (with no noise), and linear relationship corrupted with low, moderate, and high noise.

**Figure 6:**
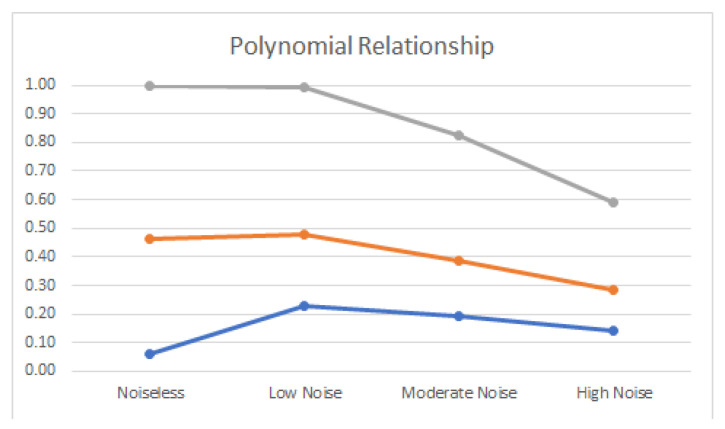
Pearson’s correlation (blue), Distance correlation (orange), and Association Factor (gray) scores for true polynomial relationship (with no noise), and polynomial relationship corrupted with low, moderate, and high noise.

**Figure 7:**
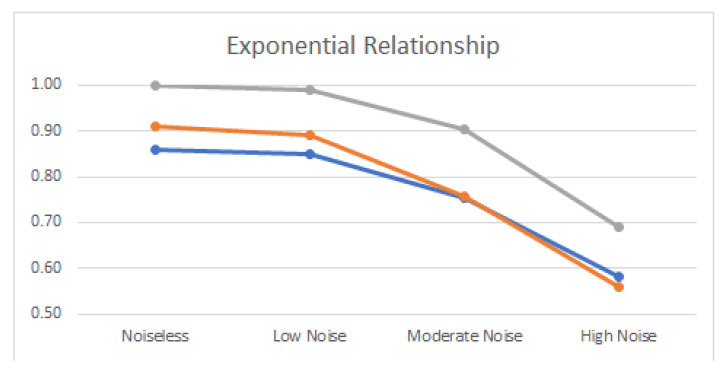
Pearson’s correlation (blue), Distance correlation (orange), and Association Factor (gray) scores for true exponential relationship (with no noise), and exponential relationship corrupted with low, moderate, and high noise.

**Figure 8:**
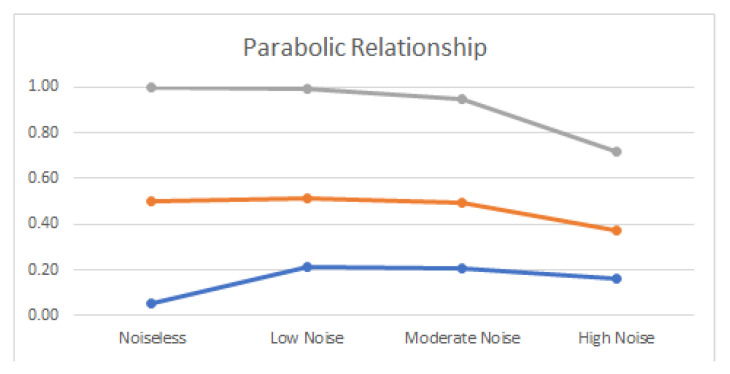
Pearson’s correlation (blue), Distance correlation (orange), and Association Factor (gray) scores for true parabolic relationship (with no noise), and parabolic relationship corrupted with low, moderate, and high noise.

Noiseless linear and nonlinear relationships are depicted in Figure 1. The noisy relationships with low, moderate, and high noise are shown in Figure 2, Figure 3 and Figure 4, respectively. The performance of Pearson’s correlation, Distance correlation, and Association Factor in identifying these linear and nonlinear relationships are depicted in Figure 5, Figure 6, Figure 7 and Figure 8. The performance of these correlation factors at different noise levels are discussed in the following section.

### 4.1. True Signal (No Noise)

Noiseless linear, exponential, parabolic, and fourth order polynomial are shown in Figure 1. Quantified correlations by Pearson’s correlation, Distance correlation, and Association Factor are summarized in Table 1. As we expect, Pearson’s correlation obtained a value of one for noiseless linear relationship, but its value was not reliable for nonlinear relationships such as exponential and polynomial. Distance correlation identified both linear and nonlinear relationships, but as we can see in Table 1, its performance was not robust with regard to the underlying relationship type between two variables. It scored one for a noiseless linear relationship, while it scored 0.47 for the fourth order polynomial, 0.91 for exponential, and 0.5 for parabolic. In contrast, the proposed AF could robustly identify the underlying relationship, and its value was one regardless of the relationship type (linear, exponential, or polynomial).

### 4.2. Noisy Relationships

Linear, exponential, parabolic, and fourth order polynomial relationships corrupted with low, moderate, and high noise are shown in Figure 2, Figure 3 and Figure 4 respectively. Pearson’s correlation, Distance correlation, and Association Factor are summarized for low, moderate, and high noise in Table 2, Table 3 and Table 4 respectively. The Pearson’s correlation absolute value dropped from one (noiseless) to 0.98, 0.82, and 0.58 for low, moderate, and high noise in identifying linear relationship between two variables. We can observe that its value is not reliable for nonlinear relationships. Its absolute value dropped from 0.86 (noiseless) to 0.58 (high noise) for exponential relationship. For fourth order polynomial, its absolute value increased from noiseless (0.06) to low noise (0.23) and then dropped from low noise to high noise (0.14). For parabolic relationship, the Pearson’s correlation absolute value increased from noiseless (0.05) to low noise (0.21) and then dropped from low noise to high noise (0.16).

Distance correlation had steady performance for linear relationship, and its value decreased from one (noiseless) to 0.56 (high noise). However, its performance for nonlinear relationships was not consistent. Its score in identifying exponential relationship was comparable with its score for linear relationship. Its value for exponential relationship was 0.98 (for noiseless) and decreased to 0.56 (for high noise). Its score for parabolic relationship was 0.50 (for noiseless) and dropped to 0.37 (for high noise). In identifying the fourth order polynomial, Distance correlation scored 0.47 for noiseless and decreased to 0.29 for high noise.

In contrast, as we can see, the proposed AF had robust performance regardless of the relationship type (Figure 5, Figure 6, Figure 7 and Figure 8). Moreover, it had robust performance in noiseless and noisy conditions. Its value for noiseless relationships (linear, exponential, parabolic, and fourth order polynomial) was steady and equal to one. Its value in low noise was still about one (0.99) regardless of the relationship type. In moderate noise condition, AF consistently identified the underlying relationship with scores from 0.82 (linear) to 0.95 (parabolic). Even in high noise condition where the underlying relationship was substantially corrupted with noise (Figure 4), AF was able to identify the underlying correlations with scores from 0.58 (linear) to 0.72 (parabolic).

AF had comparable performance with Pearson’s correlation in identifying linear relationship. It outperformed Distance correlation in identifying noiseless nonlinear relationships. Moreover, it outperformed Distance correlation in identifying nonlinear relationships in noisy conditions. Its score was up to twice (0.69) as high as Distance correlation (0.29) in identifying nonlinear correlations in high noise.

### 4.3. No Relationship

We computed Pearson’s correlation, Distance correlation, and AF for no relationship (random noise). The results are summarized in Table 5. As we can see, all correlation factors including Pearson’s correlation, Distance correlation, and AF obtained values close to zero, indicating there was no relationship between *X* and *Y*. This also clarifies that functions $h_{1}$ and $h_{2}$ did not introduce a spurious relationship into the relationship between *X* and *Y*.

### 4.4. Test of Symmetry, Sample Size, Missing Data, and Noise Level

**Figure 9:**
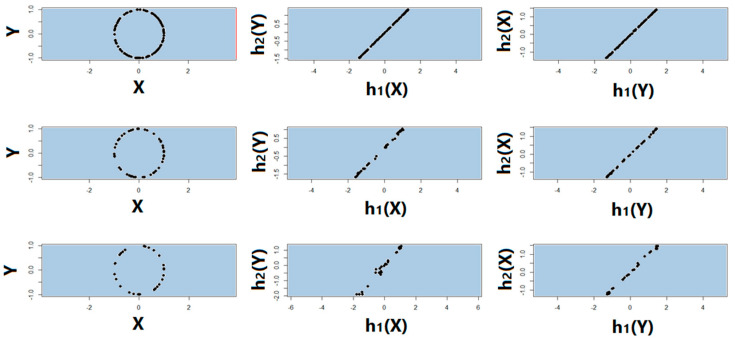
Randomly sampled true (noiseless) circular relationship. (**From top to bottom**): sample size of 100, 50, and 30 respectively; (**From left to right**): sampled true (noiseless) circular relationship; $h_{1}$ and $h_{2}$ obtained by $R_{AF}^{2}\left(X,Y\right)$; $h_{1}$ and $h_{2}$ obtained by $R_{AF}^{2}\left(Y,X\right)$.

To study the symmetry of AF quantifying the relationship between response *Y* and factor *X* regardless of their order, we computed $R_{AF}^{2}\left(X,Y\right)$ and $R_{AF}^{2}\left(Y,X\right)$ and compared them. We computed AF with regard to sample size and noise level for different relationships. Figure 9 from top to bottom shows randomly sampled true (noiseless) circular relationship of size 100, 50, and 30, respectively. The second and third columns of Figure 9 show $h_{1}$ and $h_{2}$ obtained by $R_{AF}^{2}\left(X,Y\right)$ and $R_{AF}^{2}\left(Y,X\right)$ respectively. As we can observe, the transform functions regardless of the order of the response and factor were symmetric and linear even for small sample size.

**Figure 10:**
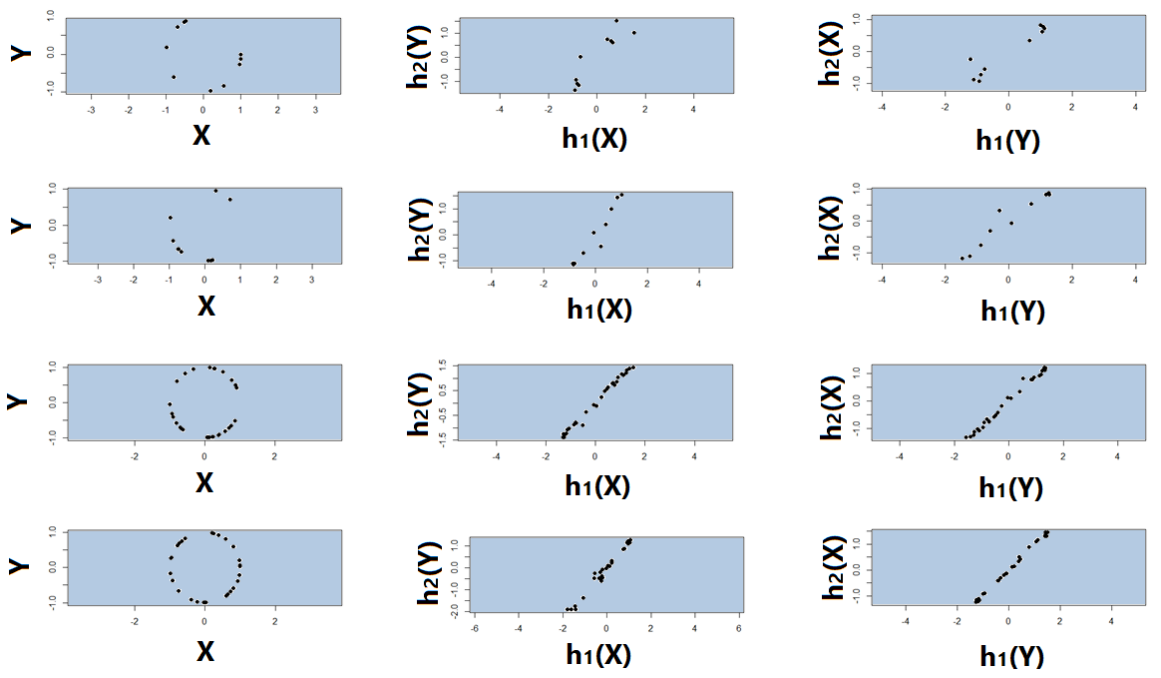
Randomly sampled true (noiseless) circular relationship with missing data (small sample size). (**First and second row**): two instances of the randomly sampled true (noiseless) circular relationship of size 10. (**Third and fourth row**): two instances of the randomly sampled true (noiseless) circular relationship of size 30. (**From left to right**): sampled true (noiseless) circular relationship; $h_{1}$ and $h_{2}$ obtained by $R_{AF}^{2}\left(X,Y\right)$; $h_{1}$ and $h_{2}$ obtained by $R_{AF}^{2}\left(Y,X\right)$.

The first and second row of Figure 10 show two instances of the randomly sampled true (noiseless) circular relationship of size 10. The third and fourth row of Figure 10 show two instances of the randomly sampled true (noiseless) circular relationship of size 30. As we can observe in this figure, because of small sample size, the true underlying relationship is not visible due to the missing data points. The second and third columns of Figure 10 show $h_{1}$ and $h_{2}$ obtained by $R_{AF}^{2}\left(X,Y\right)$ and $R_{AF}^{2}\left(Y,X\right)$ respectively. As we can observe, regardless of the order of the response and factor, and despite missing data, transform functions $h_{1}$ and $h_{2}$ were symmetric and almost linear even for a dataset with very small sample size.

Distance correlation, Maximal Information Coefficient (MIC) [24], $R_{AF}^{2}\left(X,Y\right)$, and $R_{AF}^{2}\left(Y,X\right)$ are obtained for the randomly sampled true circular relation of different sample sizes and are summarized in Table 6. As we can see, regardless of the sample size, AF could quantify the relationship even for a very small sample size of 10 (with missing data points). Moreover, AF was symmetric even for a small sample size of 30 and was almost symmetric for a very small sample size of 10. AF slightly decreased by reducing the sample size. AF outperformed MIC, and MIC performed better than Distance correlation. MIC also decreased by reducing the sample size. Distance correlation was in a range between 0.22 and 0.25 for sample sizes from 30 to 100. Its performance for sample size of 10 was sporadic. For example it scored 0.56 for a typical example of randomly sampled true circular relationship of size 10 depicted in Figure 10, second row. This could be potentially due to the arrangement of data points in this random sample of a circle that rather represents a linear relationship.

**Figure 11:**
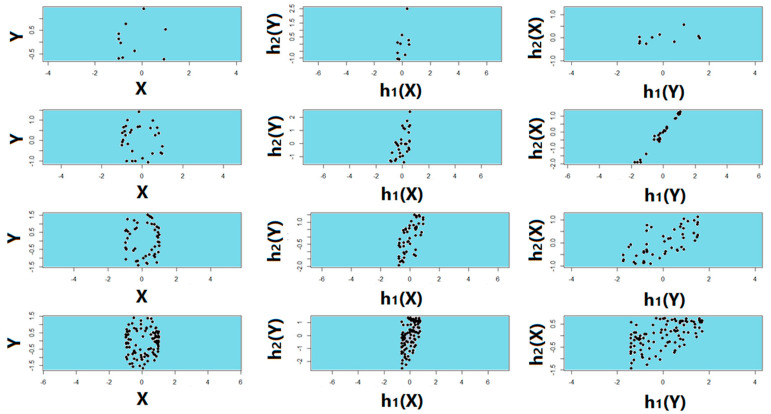
Randomly sampled circular relationship corrupted with high Gaussian noise. (**From top to bottom**): sample size of 10, 30, 50, and 100, respectively; (**From left to right**): sampled noisy circular relationship; $h_{1}$ and $h_{2}$ obtained by $R_{AF}^{2}\left(X,Y\right)$; $h_{1}$ and $h_{2}$ obtained by $R_{AF}^{2}\left(Y,X\right)$.

Figure 11 from top to bottom shows a randomly sampled circular relationship of size 10, 30, 50, and 100 respectively corrupted with Gaussian noise. The second and third columns of Figure 11 show $h_{1}$ and $h_{2}$ obtained by $R_{AF}^{2}\left(X,Y\right)$ and $R_{AF}^{2}\left(Y,X\right)$ respectively. As we can observe regardless of the order of response and factor, the transform functions were symmetric and almost linear even for a very small sample of size 10.

Distance correlation and the Maximal Information Coefficient (MIC), $R_{AF}^{2}\left(X,Y\right)$, and $R_{AF}^{2}\left(Y,X\right)$ were obtained for the randomly sampled circular relation of different sample sizes corrupted with high noise and are summarized in Table 7. As we can see regardless of the sample size, AF could quantify the relationship even for a very small sample size of 10 (with missing data points). Moreover, AF was symmetric for moderate sample size (from 50 to 100) and was almost symmetric for small and very small sample size of 30 and 10 respectively. Similar to the noiseless scenario, AF slightly decreased by reducing the sample size. AF outperformed MIC, and MIC performed better than Distance correlation. MIC values were in a range from 0.28 to 0.31 for sample size from 30 to 100, but MIC was higher for the noisy relationship with a sample size of 10. The Distance correlation values were in a range from 0.19 to 0.25 for sample size from 30 to 100; however it was 0.36 for a typical random sample of size 10 from circular relationship corrupted with noise (depicted in Figure 11).

### 4.5. Empirical Distribution of Distance Correlation, Maximal Information Coefficient, $R_{AF}^{2}\left(X,Y\right)$ and $R_{AF}^{2}\left(Y,X\right)$

**Figure 12:**
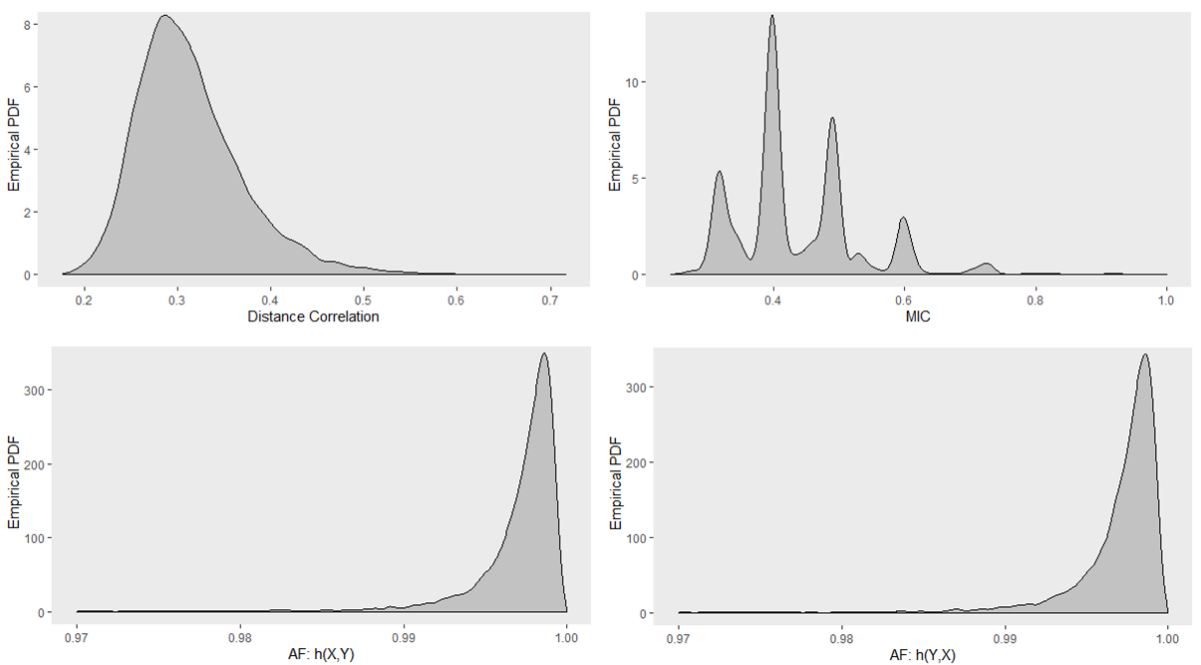
Monte Carlo empirical PDF for the true circular relationship obtained using sample size of 30. (**Top row**): Distance correlation (**left**); MIC (**right**). (**Bottom row**): Association Factor (AF) for response Y (**left**); AF for response X (**right**).

Next, we investigated the distribution of Distance correlation, Maximal Information Coefficient (MIC), $R_{AF}^{2}\left(X,Y\right)$, and $R_{AF}^{2}\left(Y,X\right)$. Figure 12 shows the Monte Carlo empirical distribution of these correlation measures for randomly sampled true circular relationship of size 30. Distributions were estimated by 1000 Monte Carlo samples. Distance correlation had a positively skewed distribution with a mode at about 0.3. MIC had a multimodal distribution with modes at about 0.3, 0.4, 0.5, and 0.6 with the highest mode at about 0.4. AF was negatively skewed with a mode at about 0.9975. We can also observe that $R_{AF}^{2}\left(X,Y\right)$ and $R_{AF}^{2}\left(Y,X\right)$ have similar distributions.

**Figure 13:**
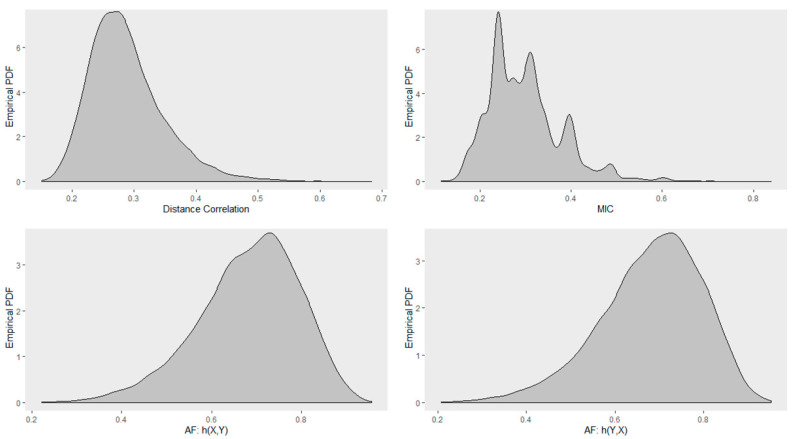
Monte Carlo empirical PDF for the circular relationship obtained using sample size of 30 corrupted with high level of Gaussian noise. (**Top row**): Distance correlation (**left**); MIC (**right**). (**Bottom row**): AF for response Y (**left**); AF for response X (**right**).

Figure 13 shows the Monte Carlo empirical distribution of these correlation measures for randomly sampled circular relationship of size 30 corrupted with high Gaussian noise. Similar to the previous simulation, distributions were estimated by 1000 Monte Carlo samples. Distance correlation had a positively skewed distribution with a mode at about 0.27. MIC had a multimodal distribution with the highest mode at about 0.25. AF was negatively skewed with a mode at about 0.7. Again here, we can see that $R_{AF}^{2}\left(X,Y\right)$ and $R_{AF}^{2}\left(Y,X\right)$ have similar distributions.

**Figure 14:**
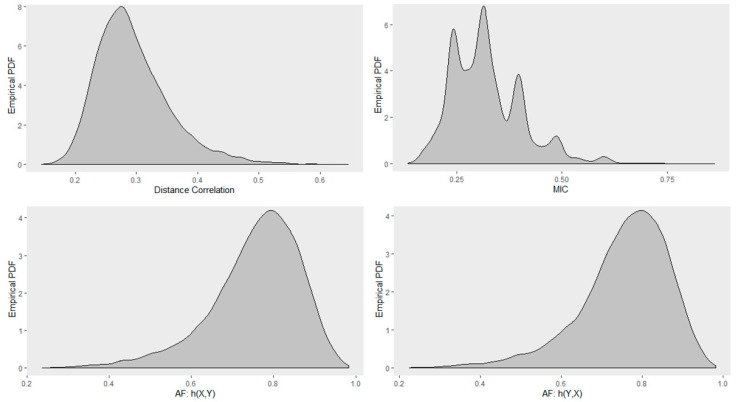
Monte Carlo empirical PDF for the circular relationship obtained using sample size of 30 corrupted with high exponential noise. (**Top row**): Distance correlation (**left**); MIC (**right**). (**Bottom row**): AF for response Y (**left**); AF for response X (**right**).

To study AF in a different noise condition, we corrupted the randomly sampled circular distribution with exponential noise and obtained the values of Distance Correlation, Maximal Information Coefficient, $R_{AF}^{2}\left(X,Y\right)$, and $R_{AF}^{2}\left(Y,X\right)$. Figure 14 shows the Monte Carlo empirical distribution of these correlation measures for randomly sampled circular relationship of size 30 corrupted with high exponential noise. Distributions were estimated by 1000 Monte Carlo samples. We see again that Distance correlation had a positively skewed distribution with a mode at about 0.27. MIC had a multimodal distribution with the highest mode at about 0.3. AF was negatively skewed with a mode at about 0.8. As we can see, $R_{AF}^{2}\left(X,Y\right)$ and $R_{AF}^{2}\left(Y,X\right)$ have similar distributions.

### 4.6. Entropic Distance

We compared the performance of Entropic Distance (ED) and the Association Factor (AF) for linear, polynomial, exponential, and parabolic relationships. Their values for noiseless, low, moderate, and high noise are summarized in Table 8. AF performed consistently with a value of one for true relationships regardless of the relationship type. ED ranged from 1.1 to about two for different true relationships. The highest value obtained by ED was for the true linear relationship. In low, moderate, and high noise, the lowest value obtained by ED was for the parabolic relationship (0.848, 0.825, and 0.812, respectively). ED did not have an upper bound and could take any positive value, while the AF values were bounded between zero and one. Therefore, rather than comparing AF and ED, we computed the AF ratio and the ED ratio in different noise conditions.

Table 9 shows the ratios as a percentage for both metrics where the noise level was increased from (a) noiseless to low noise, (b) low noise to moderate noise, and (c) moderate noise to high noise. ED ratios indicated that ED decreased by increasing the noise level. Similarly, the AF ratio decreased by increasing the noise level (Table 9). For polynomial and parabolic relationships, ED had a substantial decrease from noiseless to low noise, and it almost stabilized and had slight changes afterward by increasing the noise level. In contrast, AF had a consistent response to noise, and it decreased gradually, while its values for low noise were almost the same as the noiseless case. In comparison with ED: 1. AF is bounded; 2. AF obtains the same value regardless of the relationship type in noiseless condition; 3. AF can better quantify the correlation in noisy conditions.

### 4.7. Detrended Fluctuation Analysis

**Figure 15:**
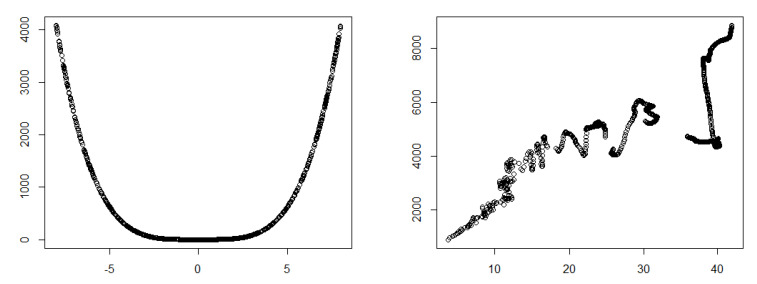
Polynomial relationship (**left**) and corresponding detrended data (**right**).

Pearson’s correlation, AF, and Detrended Fluctuation Analysis (DFA) [22] are obtained for different relationships and are summarized in Table 10. The Pearson’s correlation coefficient was computed before and after detrending the data. As we can see in the Table 10, Pearson’s correlation could identify a strong correlation even for nonlinear relationships after detrending the data. Interestingly, the DFA values were almost identical to the Pearson’s correlation values obtained for detrended data. This could be explained by visualizing the detrended data for a nonlinear relationship. As we can see in Figure 15, a polynomial relationship (Figure 15, left) was transformed to an approximately linear relationship after detrending the data (Figure 15, right). Hence, after detrending the data, Pearson’s correlation could detect the nonlinear relation. We can conclude that AF and DFA are both hybrid methods. Both methods, transform the data first, and then quantify the relationship of the transformed data.

## 5. Conclusions and Future Work

We introduced a new method to identify and quantify correlation between two variables. The proposed coefficient, Association Factor (AF), is a robust method for the identification and quantification of both linear and nonlinear associations. We applied the proposed method to several different relationships including linear, exponential, parabolic, polynomial, and circular. The results demonstrated that AF could identify both linear and nonlinear relationships. Its value was equal to one in noiseless conditions regardless of the relationship type. Moreover, we tested AF in noisy conditions where the true relationships were corrupted with noise. AF could successfully identify the correlations in low, moderate, and high noise conditions. We also tested AF under different noise distributions, Gaussian and exponential. Regardless of the noise distribution, AF could successfully quantify the correlation.

We studied the distribution of AF and compared it with the distributions of Distance correlation and MIC. We also investigated the AF values for a very small sample size where the relationship was severely under-sampled. Despite the fact that a substantial amount of data was missing due to very small sample size, AF still could quantify the underlying correlation. We compared AF with ED and discussed its advantages over ED. AF had similar performance to Pearson’s correlation in identifying linear relationship in noiseless and noisy conditions, and its value was equal to one for the noiseless linear relationship. AF outperformed Distance correlation and MIC in noiseless linear and nonlinear relationships. It also outperformed Distance correlation and MIC in noisy linear and nonlinear relationships. The results demonstrated that AF was robust with regard to the relationship type, as well as the noise condition. Although, we studied the bivariate case in this work, AF could be extended to quantify the relationship between several factors and a response, and our future work is focused on implementing the Multivariate Association Factor (MAF). The potential iterative model for a kx1 vector of factors $X_{k}$ and response *Y* can be defined by:

(19) $$\hat{h_{1}}{\left(X_{k}\right)}^{\left(t\right)}=E\left(\hat{h_{2}}{\left(Y\right)}^{\left(t-1\right)}-\hat{h_{1}}{\left(X_{k}\right)}^{\left(t-1\right)}|X_{k}\right)$$

and:

(20) $$\hat{h_{2}}{\left(Y\right)}^{\left(t\right)}=E\left(\hat{h_{1}}{\left(X_{k}\right)}^{\left(t-1\right)}|Y\right)$$

where $\hat{h_{1}}{\left(X_{k}\right)}^{\left(t\right)}$ and $\hat{h_{2}}{\left(Y\right)}^{\left(t\right)}$ are estimates of $h_{1}\left(X_{k}\right)$ and $h_{2}\left(Y\right)$ at iteration *t*, respectively. 

## Figures and Tables

**Table 1:** Pearson’s correlation, Distance correlation, and Association Factor for true relationships (without noise).

Relationship Type	Pearson’s Correlation	Distance Correlation	Association Factor
Linear	1.00	1.00	1.00
Polynomial	0.06	0.47	1.00
Exponential	0.86	0.91	1.00
Parabolic	0.05	0.50	1.00

**Table 2:** Pearson’s correlation, Distance correlation, and Association Factor for relationships with low noise.

Relationship Type	Pearson’s Correlation	Distance Correlation	Association Factor
Linear	0.98	0.98	0.98
Polynomial	−0.23	0.48	0.99
Exponential	0.85	0.89	0.99
Parabolic	−0.21	0.51	0.99

**Table 3:** Pearson’s correlation, Distance correlation, and Association Factor for relationships with moderate noise.

Relationship Type	Pearson’s Correlation	Distance Correlation	Association Factor
Linear	0.82	0.81	0.82
Polynomial	−0.20	0.39	0.83
Exponential	0.75	0.76	0.90
Parabolic	−0.21	0.49	0.95

**Table 4:** Pearson’s correlation, Distance correlation, and Association Factor for relationships with high noise.

Relationship Type	Pearson’s Correlation	Distance Correlation	Association Factor
Linear	0.58	0.56	0.58
Polynomial	−0.14	0.29	0.59
Exponential	0.58	0.56	0.69
Parabolic	−0.16	0.37	0.72

**Table 5:** Pearson’s correlation, Distance correlation, and Association Factor for no relationship.

Relationship Type	Pearson’s Correlation	Distance Correlation	Association Factor
No Relationship	0.03	0.06	0.07

**Table 6:** Distance correlation, Maximal Information Coefficient (MIC), Association Factor: $R_{AF}^{2}\left(X,Y\right)$ and $R_{AF}^{2}\left(Y,X\right)$ for the true circular relationship.

Sample Size	Distance Correlation	MIC	AF(X,Y)	AF(Y,X)
100	0.221	0.563	0.999	0.999
50	0.219	0.484	0.993	0.999
30	0.246	0.490	0.995	0.994
10	0.559	0.396	0.938	0.968

**Table 7:** Distance correlation, MIC, Association Factor: $R_{AF}^{2}\left(X,Y\right)$ and $R_{AF}^{2}\left(Y,X\right)$ for the noisy circular relationship.

Sample Size	Distance Correlation	MIC	AF(X,Y)	AF(Y,X)
100	0.205	0.282	0.579	0.579
50	0.186	0.314	0.666	0.667
30	0.249	0.304	0.573	0.536
10	0.359	0.396	0.542	0.534

**Table 8:** Entropic Distance and Association Factor for true relationships with no noise and relationships with low, moderate, and high noise.

	No Noise		Low Noise		Moderate Noise		High Noise	
Relationship Type	Entropic Distance	Association Factor	Entropic Distance	Association Factor	Entropic Distance	Association Factor	Entropic Distance	Association Factor
Linear	1.989	1.000	1.993	0.980	1.796	0.820	1.539	0.580
Polynomial	1.203	1.000	1.000	0.990	0.953	0.830	0.931	0.590
Exponential	1.845	1.000	1.847	0.990	1.749	0.900	1.512	0.690
Parabolic	1.106	1.000	0.848	0.990	0.825	0.950	0.812	0.720

**Table 9:** Changes of Entropic Distance and Association Factor in response to change of noise level.

	Noiseless to Low Noise		Low Noise to Moderate Noise		Moderate Noise to High Noise	
Relationship Types	Entropic Distance	Association Factor	Entropic Distance	Association	Entropic Distance	Association Factor
Linear	100%	98%	90%	84%	86%	71%
Polynomial	83%	99%	95%	84%	98%	71%
Exponential	100%	99%	95%	91%	86%	77%
Parabolic	77%	99%	97%	96%	98%	76%

**Table 10:** Pearson’s correlation obtained for the original and detrended data along with DFA.

Relationship Type	Pearson’s Correlation Original Data	Pearson’s Correlation Detrended Data	DFA
Linear	1	1	1
Polynomial	0.06	0.77	0.76
Exponential	0.86	0.99	0.99
Parabolic	0.05	0.88	0.88
